# Clinical Outcomes of Minced Cartilage Treatment (AutoCart™) for Medial Osteochondral Lesions of the Talus: A Prospective One-Year Follow-Up Study

**DOI:** 10.3390/jcm14248710

**Published:** 2025-12-09

**Authors:** Klaus E. Roth, Gian M. Salzmann, Philipp Winter, Irene Schmidtmann, Gerrit Maier, Isabelle Cochrane, Robert Ossendorff, Kajetan Klos, Philipp Drees

**Affiliations:** 1 Orthopaedics and Orthopaedic Surgery, Meliva Gelenkzentrum Rheinmain, 65239 Hochheim, Germany; 2 Department of Orthopaedics and Orthopaedic Surgery, Saarland University Medical Center, 66421 Homburg, Germany; 3 Institute of Medical Biostatistics, Epidemiology and Informatics, University Medical Centre of the Johannes Gutenberg, University Mainz, 55131 Mainz, Germany; 4 Rehazentrum am Meer, Carl von Ossietzky University Oldenburg, Unter den Eichen 18. 26160 Bad Zwischenahn, 26129 Oldenburg, Germany; 5 Department for Orthopaedics and Trauma, University Hospital Bonn, 53127 Bonn, Germany; 6 School of Medicine, University of St Andrews, St Andrews KY16 9TF, UK; 7 Department of Orthopedics and Traumatology, University Medical Center of the Johannes Gutenberg University Mainz, 55131 Mainz, Germany

**Keywords:** minced cartilage implantation, cartilage regeneration, talus, ankle

## Abstract

**Background/Objectives:** This prospective study aims to assess the clinical outcomes of the AutoCart™ technique for the treatment of medial osteochondral lesions of the talus (OLT). **Methods:** 29 consecutive patients treated for medial OLT were included. Demographic characteristics and preoperative imaging (MRI and CT) were reviewed, and patient-reported outcome measures (PROMs)—including a Visual Analog Pain Scale (VAS), Foot Function Index (FFI), Foot and Ankle Ability Measure (FAAM), and the Veterans RAND 12-Item Health Survey (VR-12)—were assessed preoperatively and at 6 and 12 months post-treatment. **Results:** In the cohort, 14 (48%) were female, 13 (45%) were male, and 2 (7%) did not disclose their gender. Median age was 35.5 years (interquartile range: 23.0–49.5). Mean defect size was 121.95 ± 84.46 mm^2^. Three patients were treated entirely arthroscopically, while 26 patients underwent medial malleolar osteotomy with cancellous bone grafting from the calcaneus for cartilage fragment placement. At one-year follow-up, there were significant improvements in pain and functional outcomes. The VAS score showed a mean reduction of 1.3 points (95% CI: −2.6 to −0.1; *p* = 0.036). Strong improvement was observed in the FFI, with a mean reduction of 13.3 points (95% CI: −21.0 to −5.6; *p* = 0.001). The FAAM Sports subscale showed a significant increase of 18.6 points (95% CI: 7.0 to 30.1; *p* = 0.002). **Conclusions:** Patients demonstrate clinical improvement after minced cartilage implantation with the AutoCart™ technique. These findings suggest that the minced cartilage procedure is a viable treatment option for medial OLTs, though further studies are needed to assess long-term efficacy.

## 1. Introduction

Osteochondral lesions of the talus (OLTs) are defined by damage to the articular cartilage of the talus and the underlying subchondral bone. Traumatic events are considered the primary cause, with up to 75% of OLTs occurring following injury [1,2,3]. Altered biomechanical properties of hyaline cartilage under abnormal load can contribute to the development of chondral lesions [4], while concomitant injury to the subchondral bone plate significantly increases the risk of cystic degeneration or osteonecrosis of the talus [5].

Currently, the preferred management strategy for OLTs is an individualized treatment algorithm based on patient- and lesion-specific characteristics of both bone and cartilage [6]. There is currently no well-defined gold standard available. Conservative management comprises partial weight bearing, physiotherapy and the prescription of nonsteroidal anti-inflammatory drugs. Local infiltrations with corticosteroids or hyaluronic acid are also often performed in symptomatic patients. Nevertheless, these treatment approaches are only successful in approximately 50% of cases [7]. Established surgical strategies are generally classified into three categories: cartilage repair, cartilage replacement, and cartilage regeneration [8], with reported success rates ranging from 76% to 89% [7,9]. Among these, the most frequently performed techniques are curettage and bone marrow stimulation (BMS), osteochondral autograft transplantation (OATs), autologous chondrocyte transplantation with bone augmentation (ACI), transmalleolar drilling and fixation and the implantation of osteochondral allografts. To date, no single surgical technique has proven superior, and there is no consensus on the optimal treatment algorithm.

Minced cartilage offers a promising approach for reliably reconstructing hyaline cartilage architecture, without the morbidity and technical challenges associated with other restorative techniques such as osteochondral grafting or autologous chondrocyte implantation (ACI) [10]. The technique involves fragmenting cartilage tissue to increase its surface area, thereby promoting chondrocyte proliferation—a process referred to as “activation” [11]. This leads to cellular outgrowth and coverage of the defect with newly formed chondral tissue. Schneider et al. demonstrated that minced cartilage implantation in the knee produced favorable outcomes after two years, with continuous clinical improvement observed between 3 and 24 months postoperatively [12]. The current evidence has a strong focus on the knee, while results of MCI treatment of OLTs of the talus are not widely reported in the literature.

The present study aims to evaluate the clinical outcomes of minced cartilage implantation in the ankle joint at one year follow up.

## 2. Materials and Methods

### 2.1. Ethical Approval

The ethical approval for this study was granted by the Ethics Committee of the State Medical Association of Hesse under processing number 2021-2546-evBO.

### 2.2. Cohort Selection

Patients with persistent pain and impaired function of the ankle and diagnosed osteochondral lesion of the talus who met the eligibility criteria were included in the study. The inclusion and exclusion criteria are described in Table 1. Prior to undergoing surgical intervention, all patients received a six-month course of conservative therapy, either administered by the authors’ institution or, in the case of external referrals, by the referring physician in accordance with the recommendations of the International Consensus Meeting on Cartilage Repair of the Ankle.

### 2.3. Radiological Assessment

**Figure 1:**
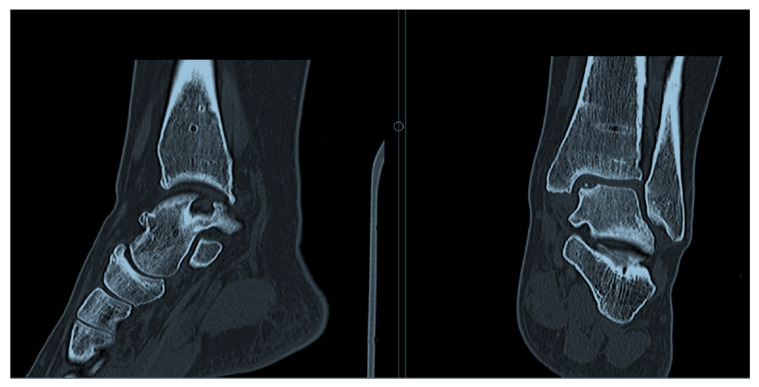
Representative CT scan sagittal and coronal view of a medial osteochondral lesion of the talus prior to treatment with AutoCart™.

**Figure 2:**
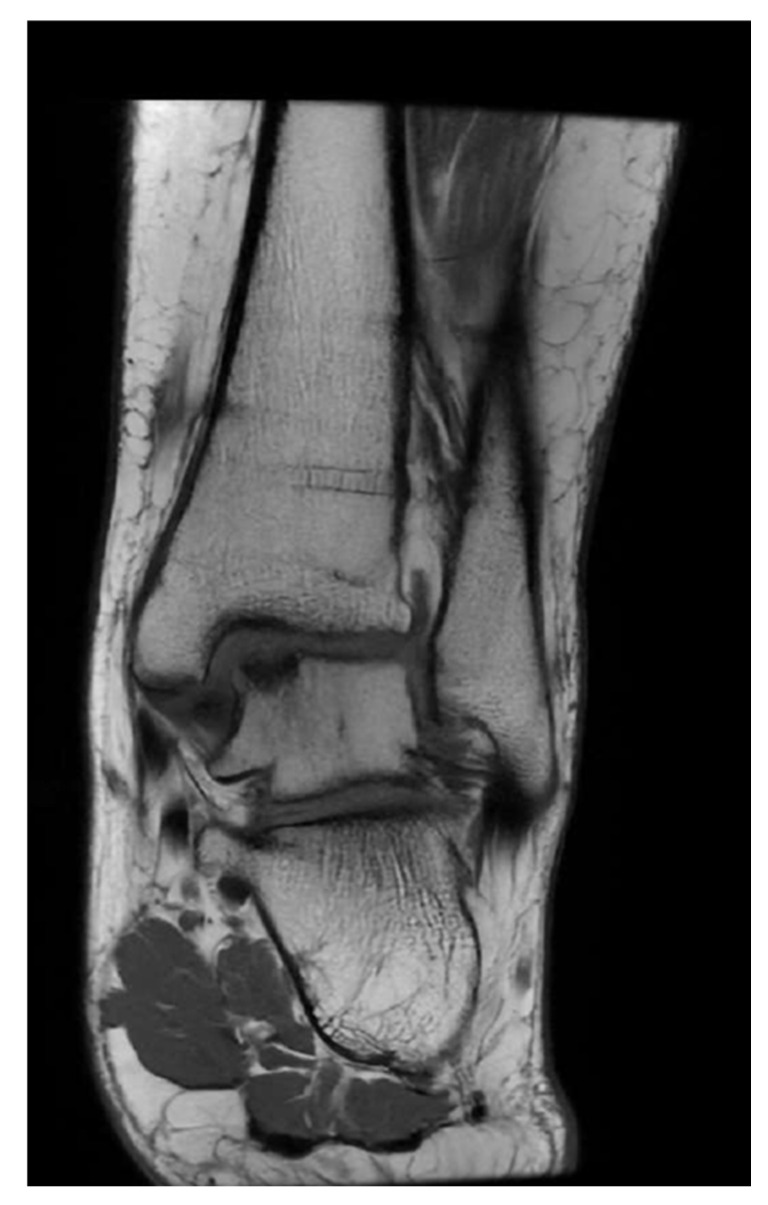
Representative MRI scan (coronal view, T1 sequence) of a medial osteochondral lesion of the talus prior to treatment with AutoCart™.

Lesion location and size were evaluated using CT imaging for cases involving bony defects (Figure 1) and T1-weighted MRI for those without osseous involvement (Figure 2). Following initial diagnostic imaging, all patients underwent preoperative computed tomography (CT, Siemens SOMATOM Definition AS, Munich, Germany) to assess the extent and morphology of the osseous defect in the talus. Preoperative CT imaging was performed by a single radiologist with a standardized protocol for assessing defect size due to its superior accuracy compared to MRI [13]. Imaging was performed using a multi-detector CT scanner (e.g., 64-slice system), with patients positioned supine and the affected ankle placed in a neutral position. Thin-slice axial images (≤1.0 mm slice thickness) were acquired from the distal tibia to the calcaneus, with multiplanar reconstructions (axial, coronal, sagittal) performed to allow for detailed three-dimensional evaluation of the defect. Bone algorithm reconstruction was applied to enhance visualization of cortical and subchondral bone integrity. There is no standardized lesion morphology reporting [13]; on this occasion, lesions were described through volumetric assessment using the ellipsoid formula [14]. Lesion dimensions were measured in the coronal plane on the medial aspect of the talus, determining the maximum coronal diameter and depth using electronic markers within the PACS system. The same measurements were obtained in the sagittal plane for the maximum sagittal diameter [15]. The surface area of elliptical osteochondral defects was calculated using the standard ellipse area formula: A = π ⋅ a ⋅ b, where ‘a’ is half of the maximum coronal diameter (semi-major axis) and ‘b’ is half of the maximum sagittal diameter (semi-minor axis). This approach offers a geometrically accurate approximation of the affected articular surface, particularly for lesions with an elliptical contour [16]. Furthermore, lesions were classified according to the revised Hepple et al. classification system [17]. We assessed both intra- and inter-rater reliability for all radiological parameters, including coronal and sagittal diameter, vertical depth, and defect area. Two independent musculoskeletal surgeons evaluated all CT scans. To assess intra-rater reliability, a subset of cases *(n* = 29) was re-evaluated by both surgeons after an interval of 3 weeks. Inter-rater reliability was calculated using the intraclass correlation coefficient (ICC) for continuous variables, yielding a value of 0.79, which indicates good agreement. Although intra-rater reliability was not formally calculated, the repeated assessments after a 3-week interval also reflect consistency in image interpretation under blinded conditions.

### 2.4. Surgical Procedure

Initially, a diagnostic arthroscopy of the affected ankle joint was performed in all patients. Unstable cartilage from the osteochondral lesion of the talus (OLT) was harvested as donor tissue for the AutoCart™ procedure (Arthrex Inc., Naples, FL, USA), following previously described protocol [18]. Autologous conditioned plasma (ACP) was prepared according to the manufacturer’s instructions (Arthrex, Naples, FL, USA) and processed using the Thrombinator™ (Arthrex, Naples, FL, USA) to generate thrombin solution.

**Figure 3:**
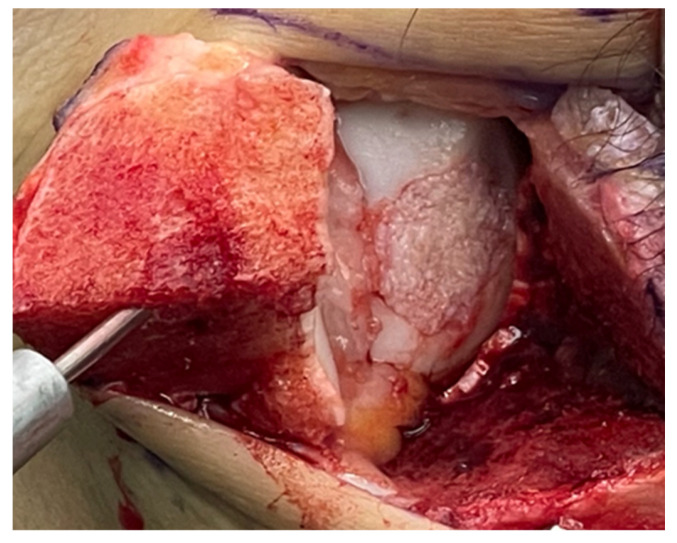
Intraoperative view following medial malleolar osteotomy and mobilization of the medial malleolus (visible on the left side of the image). The osteochondral defect of the medial talar dome is clearly exposed. Minced cartilage fragments, with a paste-like configuration, have been evenly distributed across the depth of the prepared defect bed.

Cartilage grafts were arthroscopically collected using a 3.0 mm × 7 cm Sabre, SJ (Arthrex, Naples, FL, USA), and captured with the Graftnet™ (Arthrex, Naples, FL, USA), irrespective of whether the subsequent cartilage chip transplantation was performed via an open or arthroscopic approach. The harvested cartilage was processed to achieve a homogeneous, paste-like graft consistency. Prior to application, the cartilage chips were mixed with a few drops of ACP. For arthroscopic placement of the chips, a Tuohy needle, Short with Obturator (Arthrex, Naples, FL, USA), was utilized. Autologous thrombin was applied over the cartilage chips, and in the final step, a mixture of thrombin and ACP was applied to the construct, sealing the defect with autologous fibrin glue (Figure 3).

If sclerosis of the defect bed was suspected during debridement, antegrade perforation was performed using a 1.0 mm Kirschner wire.

In alignment with the International Consensus Group’s guidelines, bone grafting (impaction of allogeneic cancellous bone) was considered when intraoperative debridement revealed subchondral bone loss exceeding 3 mm [19]. In such cases, the defect zone was exposed via a V-shaped medial malleolar osteotomy, with AutoCart™ transplantation following atop the bone graft (Figure 1). All procedures were executed by two experienced foot and ankle surgeons (KER, KK). For patients presenting with lateral instability, additional autologous ligament reconstruction was performed.

### 2.5. Modified Broström-Gould Procedure

A curvilinear incision was made over the distal fibula to expose the anterior talofibular ligament (ATFL) and, if necessary, the calcaneofibular ligament (CFL). Both ligaments were anatomically repaired using non-absorbable sutures or suture anchors at their fibular insertion. The repair was reinforced by advancing the inferior extensor retinaculum over the lateral capsule and securing it to the fibula (Gould modification). Stability was assessed clinically intraoperatively.

### 2.6. Postoperative Management

Postoperatively, the ankle was immobilized in a VACO^®^ped boot (OPED, Valley/Oberlaindern, Valley, Germany) in a functional position of 0° dorsal extension of the ankle. Partial weightbearing of up to 20 kg was permitted for 6 weeks, supported by the use of forearm crutches. Despite immobilization, pain-adapted active range of motion was encouraged from day one to support joint mobility and cartilage nutrition. Progressive weightbearing and physiotherapy were initiated based on clinical progress and imaging findings. Full weightbearing was achieved at 3 months on average. Proprioceptive training was a key element of physiotherapy-assisted rehabilitation, to ensure full control during movement was gained.

### 2.7. Outcome Variables

In addition to general demographic data, the clinical condition of the patients was assessed using the Foot Function Index (FFI), Visual Analog Pain Scale (VAS), Foot and Ankle Ability Measure (FAAM), and VR12 questionnaires. Patients received automated email invitations at predefined time points (preoperatively, as well as at 6 and 12 months postoperatively) to complete validated questionnaires online. The VAS scale was additionally assessed 2 weeks and 6 weeks postoperatively.

### 2.8. Data Collection

Patient-reported clinical outcomes were systematically collected using the Surgical Outcomes System (SOS, Arthrex Inc., Naples, FL, USA), a web-based, GDPR-compliant platform designed for the longitudinal assessment of Patient-Reported Outcome Measures (PROMs).

### 2.9. Statistics

Categorical variables were summarized by absolute and relative frequencies. Quantitative variables were summarized by mean, standard deviation, median, minimum, maximum and quartiles. To describe how patient reported outcomes evolved over time, these were displayed in boxplot by follow-up visit. The significance of changes in patient reported outcomes (while taking age into account) was assessed using a linear mixed model with visit and age as fixed effect and patient as random effect. The significance level was chosen as α = 0.05. No correction for multiple testing was performed as the study was observational and the analysis therefore exploratory.

## 3. Results

Between April 2022 and January 2023, 43 consecutive patients were screened, 39 of whom fulfilled the inclusion criteria (Figure 2). 9 did not provide informed consent and 1, while providing informed consent, chose not to fill in any questionnaires; thus, 29 patients with evidence of a medial talus osteochondral lesion were included in the study.

### 3.1. Demographics and Patient Characteristics

**Figure 4:**
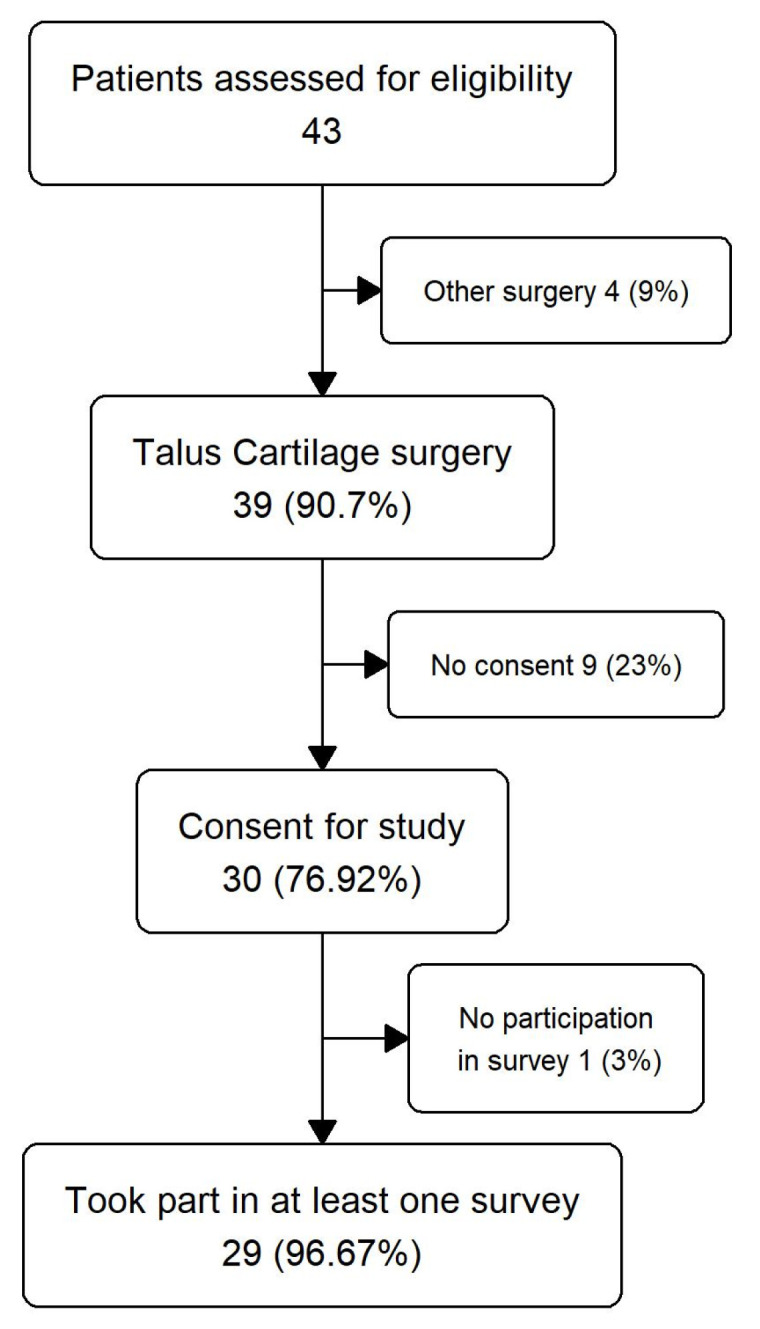
Flow chart of study participation.

During the treatment and follow-up period, no adverse or serious adverse events occurred. A total of 29 patients completed the pre-treatment questionnaires at least partially (Figure 4). Of these, 14 (48%) were female, and 15 (52%) were male (Table 2). Mean age (SD) was 36.9 (16.3). A total of 97% of the defects were located in the central or posteromedial region of the talar dome, and 93% had a surface area greater than 100 mm^2^ (Table 3 and Table 4). Only three patients were treated using an entirely arthroscopic technique (Table 4). In all remaining cases, minced cartilage fragments were harvested arthroscopically but implanted via a medial malleolar osteotomy following cancellous bone grafting (Supplementary Table S1). In 16 cases, a concomitant lateral ligament reconstruction was performed (Table 4).

### 3.2. Patient-Reported Outcome Measures (PROMs)

**Figure 5:**
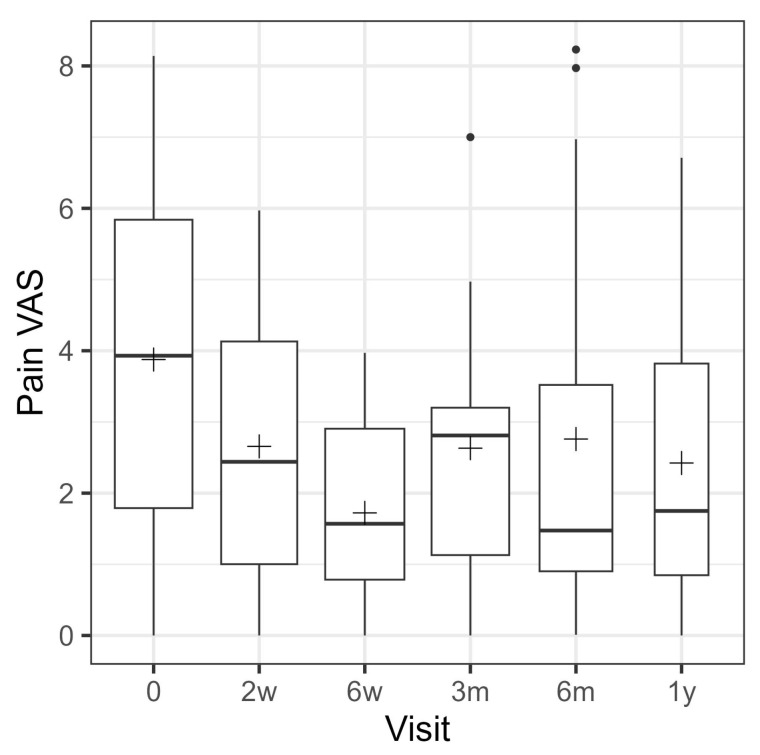
Pain VAS development over time.

Pre-treatment pain, as assessed by the Visual Analog Pain Scale (VAS), was reported by 25 patients, with a mean (SD) score of 3.9 (2.4) (Figure 5). Female patients (*n* = 12) reported a higher mean pain VAS of 5.0 (2.2), compared to male patients (*n* = 13), who reported a mean pain VAS of 2.8 (2.2). The pre-treatment VR-12 physical score was reported by 25 patients, with a mean score of 38.6 (9.4). Female patients (*n* = 12) had a lower mean score of 35.6 (8.2), compared to male patients (*n* = 13), who reported a mean of 41.4 (9.8). The pre-treatment VR-12 mental score was also reported by 25 patients, with a mean of 45.5 (9.5). Female patients (*n* = 12) had a mean of 45.9 (7.6), while male patients (*n* = 13) reported a mean of 45.1 (11.3), showing similar mental health scores across genders. The Foot Function Index was similar for both genders, with women (*n* = 12) reporting a mean of 34.6 (10.2) and men (*n* = 13) reporting a mean of 32.8 (13.3). The overall mean across 25 patients was 33.6 (11.7). The FAAM sports subscale was reported by 12 women, with a mean of 28.6 (20.9), which was somewhat lower than the mean of 34.1 (20.3) reported by 13 men. Overall, 25 patients reported a mean of 31.5 (20.3) on the FAAM sports subscale.

With respect to the mean VAS pain score, interval time conferred a statistically significant effect (*p* = 0.001). Mean VAS pain was significantly lower than pre-treatment at all follow-up visits. The most significant improvement was observed at 6 weeks, with an average difference of −2.2 (95% CI = −3.2, −1.3, *p* < 0.001). At the one-year follow-up, the difference was −1.2 (95% CI = −2.6, −0.1, *p* = 0.036). At baseline, the mean VR-12 physical score was 38.6 (9.4) (Figure 4). There was no significant difference in scores between visits (*p* = 0.104). The average difference at the one-year follow-up was 5.2 (95% CI = −0.9, 11.3, *p* = 0.090).

**Figure 6:**
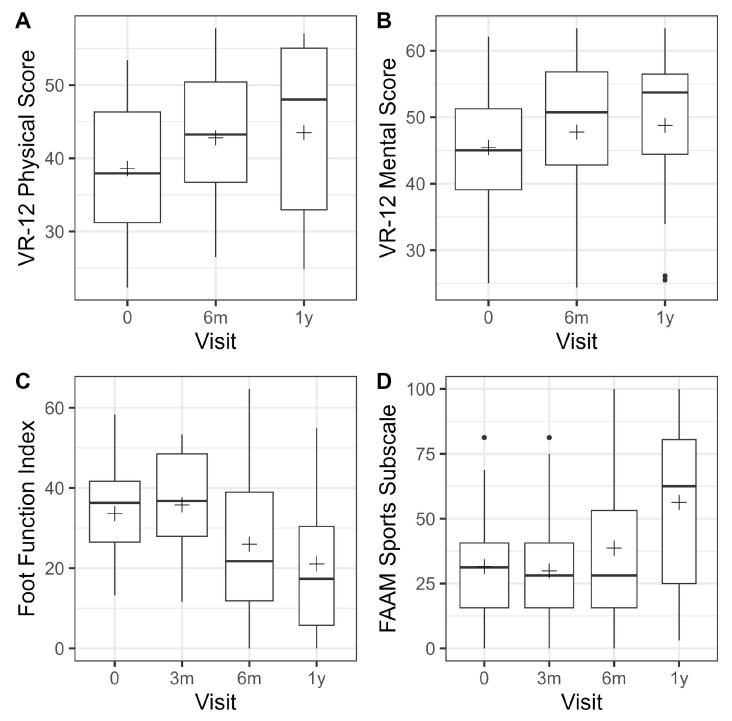
Development of patient reported outcomes over time. Box plots (**A**) VR-12 Physical Score, (**B**) VR-12 Mental Score, (**C**) Foot Function Index, (**D**) FAAM Sports Subscale. The box plots depict the patient reported outcomes by follow-up visit. The width of the box plots depicts the sample size available at the corresponding follow-up visit.

Pre-treatment, the mean VR-12 mental score was 45.5 (9.5) (Figure 6). No significant difference in scores was observed between visits (*p* = 0.136). The average difference at the one-year follow-up was 4.8 (95% CI = −1.0, 10.5, *p* = 0.100). Pre-treatment, the mean Foot Function Index was 33.6 (11.7). A significant effect of visit was observed (*p* < 0.001). The mean Foot Function Index was significantly lower than pre-treatment at both the 6-month and 1-year follow-up visits. The most pronounced effect was observed at 1 year, with a difference of −13.3 (95% CI = −21.0, −5.6, *p* = 0.001). The mean FAAM sports subscale score was 31.5 (20.3) at baseline. A significant effect of visit was observed (*p* = 0.003). The mean FAAM sports subscale score was significantly higher than pre-treatment at the 1-year follow-up, with a difference of 18.6 (95% CI = 7.0, 30.1, *p* = 0.002).

The box plot depicts pain VAS at baseline, 2 weeks, 6 weeks, 3 months, 6 months and 1 year after surgery. The width of the box plots depicts the sample size available at the corresponding follow-up visit (Table 5).

This table displays the estimated mean changes in the patient reported outcomes obtained from the linear mixed models, together with 95% confidence intervals and corresponding *p*-values. Only the visual analog pain scale was recorded at each visit.

## 4. Discussion

One-year follow-up of patients with medial osteochondral lesions of the talus (OLTs) treated with AutoCart™ demonstrated promising clinical outcomes. Significant improvements were observed in three of five patient-reported outcome measures (VAS, FFI, FAAM-Sports subscale). The VAS score showed a small but statistically significant reduction in mean score, with a more pronounced improvement with respect to the FFI and FAAM Sports subscale. Additionally, both VR-12 Physical and Mental Component Summary scores increased at one year compared to baseline, though these changes did not reach statistical significance—possibly due to the limited sensitivity of these instruments to surgical intervention, or the small sample size. Recent data suggest that pathologies of the cartilage confer a mental health burden, on which treatment has a significant impact. Our findings align with this evidence [19].

Pain reduction occurred relatively early, whereas functional improvements progressed more gradually. The statistical model revealed a continuous improvement trajectory, with VAS scores decreasing six months and at one year. Similarly, the FAAM Sports subscale showed a modest initial improvement (6.8 points at six months), increasing to 18.6 points at the one-year mark. A follow-up period of 1 year is relatively short, especially for the assessment of functional outcome measures, and as such, may not paint a full picture of the improvements that can be expected.

The observed gradual recovery likely reflects the biological processes underlying cartilage regeneration. Chondrocytes embedded in a biologically active matrix proliferate and gradually form new cartilage through cellular outgrowth [20]. These regenerative mechanisms involve time-dependent phases, including cell proliferation, extracellular matrix deposition, and tissue remodeling [21]. Early postoperative times are characterized by immature repair tissue, which may explain the delay in functional recovery. Optimal outcomes, which are likely attributable to the gradual maturation of the tissue, typically become apparent around one year postoperatively [22].

Direct comparison of our results with existing literature is limited, as this is the only prospective study investigating minced cartilage treatment for talar lesions [23]. The only available study on minced cartilage, which describes the same technique employed in our study, is of a retrospective design, and is limited by a small sample size (*n* = 9), the involvement of multiple surgeons, and the absence of the paste-like configuration of cartilage fragment, which is critical for achieving surgical success, leading to a revision rate > 50% [24]. In a retrospective analysis involving 32 patients, Shim described cartilage mincing using a scalpel (1 mm^3^ according to the study) and fixation of the fragments with fibrin glue. Although this method did not achieve a paste-like consistency, a 67% improvement in the Foot Function Index (FFI) was reported [25].

Studies that demonstrate comparable methods were performed in particulate juvenile cartilage [10]. The average clinical scores reported approximately one year postoperatively were similar to those achieved with other established treatments, including bone marrow stimulation [26,27]. However, this technique appears to be effective primarily for smaller lesions, whereas larger defects—as were predominantly observed in our study—are associated with poorer outcomes.

In Europe, acellular scaffold augmentation remains the most commonly used technique for treating osteochondral defects exceeding 100 mm^2^ [28]. In our cohort, which predominantly included lesions exceeding 100 mm^2^, clinical outcomes as measured by the Foot Function Index (FFI) were comparable to those reported for scaffold-based techniques [29]. Following minced cartilage treatment, FFI scores improved by 62%, closely aligning with results in scaffold augmentation from Gottschalk et al. (59% improvement) [29] and Walther [30] who observed a 54% reduction in pain and a 53% improvement in function. Kubosch et al. [31] reported a mean post-treatment FFI score of 34, which is consistent with the outcomes observed in our study. However, the considerable variability in defect sizes across the aforementioned studies limits direct comparability—not only between the scaffold-based studies themselves, but also in relation to our own study.

Most lesions in our study were located centrally or posteromedially on the talar dome and exceeded 100 mm^2^. According to international guidelines, lesions of this size warrant augmentation [28] suggesting that microfracture alone would have been insufficient. One debated aspect in treatment of large OLTs is whether drilling of the subchondral bone beneath implanted cartilage is beneficial. Drilling may promote revascularization and mesenchymal stem cell release, leading to fibrocartilage formation [32]. However, adverse effects—such as subchondral bone plate depression—have also been reported and have been associated with worse outcomes [33]. Reilingh et al. found bone plate depression in 79% of BMS patients at one-year follow-up [34]. Based on these findings, a different approach was adopted in the present study. Drilling was performed only in cases where pronounced sclerosis of the defect bed was identified intraoperatively. In all other patients, perforation was deliberately avoided in order to preserve the structural integrity of the subchondral bone plate and potentially reduce the risk of postoperative subsidence. Larger prospective studies with matched cohorts, standardized treatment protocols, consistent outcome reporting, and clearly defined inclusion criteria, outcome measures, rehabilitation protocols, and return-to-sport timelines are required [35].

### Limitations

This study is limited by its small cohort (*n* = 29) and follow-up attrition. No significant differences in baseline demographics or patient reported outcome scores were observed between patients who completed the follow-up and those who did not. Patients who answered the 1-year questionnaire and those who did not answer did not differ significantly with respect to the Pain VAS, the VR-12 physical and mental scores, and the foot function index at intermediate time points. Patients who answered the 1 year questionnaire had on average higher values in the FAAM-sports subscale, but the only significant difference was found at 6 months. Thus, the loss to follow-up may have been influenced somewhat by patient outcomes. As patients who completed the follow-up had higher values in the FAAM-sports it could be argued that the results are over-optimistic. The short data collection period also limits generalizability. No correlation analysis between defect size, its location and clinical outcomes was conducted due to the limited patient sample, and the generally high uniformity of defect dimensions. Similarly, the small sample size prevented meaningful analysis of demographic variables or secondary predictors, including defect-specific factors (size, location, containment), patient characteristics (age, BMI, activity level), surgical techniques, and rehabilitation protocols. In the study, 16 patients underwent simultaneous ligament reconstruction. Moreover, the authors describe a high incidence of medial osteotomy, but there is no analysis of the possible impact of osteotomy on joint function. There is no analysis of the impact of this procedure on pain and function available. Although current evidence suggests that medial malleolar osteotomy does not significantly affect clinical outcomes [36], its impact cannot be entirely ruled out. Postoperative MRI was not performed, as MRI- (MOCART [37]) scores do not reliably correlate with clinical outcomes; findings such as edema, cartilage irregularities, and bone changes have unclear clinical significance [38]. Evaluating such aspects with uncertain bearing on clinical success did not seem appropriate in the context of this study. The limitations of using only clinical PROMs without objective biomechanical measurements or post-operative imaging (MRI/MOCART) should be taken into account, even if the latter do not fully correlate with the outcome.

## 5. Conclusions

In this prospective case series of 29 patients with medial OLTs, AutoCart™ demonstrates improved clinical outcomes in terms of pain reduction and gain of function within one year postoperatively. These findings suggest that minced cartilage may offer an effective treatment option for medial OLTs, though further studies are needed to assess long-term efficacy.

## Figures and Tables

**Table 1:** Inclusion and Exclusion criteria.

Inclusion Criteria	Exclusion Criteria
Age > 18 years Full-thickness chondral and osteochondral defects in the medial talar shoulder Symptoms (pain, loss of function) Conservative therapy-resistant course > 6 months Traumatic and non-traumatic lesions	Age < 18 years Rheumatic diseases Metabolic-associated cartilage damage (e.g., gout) Infection Body mass index > 34 kg/m^2^ Pregnancy Inability to comply with postoperative instructions Prior Surgeries of the ankle Revision procedures

**Table 2:** Demographics and baseline characteristics of patients with completed questionnaires, stratified by Gender.

	Sex		
Characteristic	F *n* = 14 ^1^	M *n* = 15 ^1^	*p*-Value ^2^
*Age at treatment*			0.7
N Non-missing	14	15	
Mean (SD)	35.3 (16.7)	38.4 (16.3)	
*Body height [cm]*			0.021
N Non-missing	12	11	
Mean (SD)	166.0 (10.5)	175.4 (7.7)	
*Body weight [kg]*			0.10
N Non-missing	12	11	
Mean (SD)	73.4 (16.1)	85.7 (11.9)	
*Body mass index (BMI)*			0.5
N Non-missing	12	11	
Mean (SD)	26.5 (4.8)	27.9 (4.0)	
*Body mass category*			0.9
normal weight	5 (42%)	3 (27%)	
Overweight	4 (33%)	5 (45%)	
Obese	3 (25%)	3 (27%)	
*Pre-treatment: Visual Analog Pain Scale: Visual Analog Pain Scale*			0.016
N Non-missing	12	13	
Mean (SD)	5.0 (2.2)	2.8 (2.2)	
*Pre-treatment: VR12: VR-12 Physical Score*			0.14
Mean (SD)	35.6 (8.2)	41.4 (9.8)	
*Pre-treatment: VR12: VR-12 Mental Score*			>0.9
Mean (SD)	45.9 (7.6)	45.1 (11.3)	
*Pre-treatment: Foot Function Index: Foot Function Index*			0.9
Mean (SD)	34.6 (10.2)	32.8 (13.2)	
*Pre-treatment: FAAM—Sports Subscale: FAAM Sports Subscale*			0.4
Mean (SD)	28.6 (20.9)	34.1 (20.3)	

^1^ *n* (%). ^2^ Wilcoxon rank sum test; Wilcoxon rank sum exact test; Fisher’s exact test.

**Table 3:** Lesion size measurements.

Measuring Range	Result (*n* = 29)
Coronal diameter (mm) (Mean ± SD)	9.07 ± 2.01
Sagital diameter (mm) (mean ± SD)	13.67 ± 3.94
Vertical depth (mm) (mean ± SD)	7.73 ± 1.03
Area (mm^2^) (mean ± SD)	121.95 ± 54.46
<100 mm^2^	2
≥100 mm^2^	27

**Table 4:** Surgical procedures.

Item	Number (*n* = 29)
Malleolar osteotomy	26
Purely arthroscopic	3
Concomitant ligament reconstruction	16
Bone plasty	26

**Table 5:** Results of linear mixed model—Changes in patient reported outcomes compared to baseline.

		Visual Analog Pain Scale			VR-12 Physical Score			VR-12 Mental Score			Foot Function Index			FAAM—Sports Subscale		
Time	*n*	Mean	95% CI	*p*-Value	Mean	95% CI	*p*-Value	Mean	95% CI	*p*-Value	Mean	95% CI	*p*-Value	Mean	95% CI	*p*-Value
2 w	24	−1.2	[−2.2; −0.2]	0.0153												
6 w	26	−2.2	[−3.2; −1.3]	<0.0001												
3 m	25	−1.3	[−2.3; −0.3]	0.0110							1.3	[−4.6; 7.3]	0.6545	−1.6	[−10.6; 7.4]	0.7168
6 m	22	−1.2	[−2.3; −0.2]	0.0177	4.5	[−0.5; 9.5]	0.0777	4.2	[−0.5; 8.9]	0.0801	−9.1	[−15.4; −2.7]	0.0057	6.8	[−2.7; 16.4]	0.1551
1 y	12	−1.3	[−2.6; −0.1]	0.0357	5.2	[−0.9; 11.3]	0.0904	4.8	[−1.0; 10.5]	0.1000	−13.3	[−21.0; −5.6]	0.0011	18.6	[7.0; 30.1]	0.0021

## Data Availability

The original contributions presented in this study are included in the article/Supplementary Material. Further inquiries can be directed to the corresponding author.

## Supplementary Materials

The following supporting information can be downloaded at: https://www.mdpi.com/article/10.3390/jcm14248710/s1, Table S1: Comparison of patients with and without one-year follow-up.

## Abbreviations

The following abbreviations are used in this manuscript:

ACI	Autologous Chondrocyte Implantation
CT	Computer tomography
FAAM	Foot Ankle Ability Measure
FFI	Foot Function Index
MCI	Minced Cartilage Implatation
MRI	Magnete Resonance Imaging
OATs	Osteochondral autograft transplantation
SOS	Surgical Outcome System
VAS	Visual analogue scale
VR 12	Veterans RAND 12-Item Health Survey
