# Supplementary material for: Clinical Outcomes of Minced Cartilage Treatment (AutoCart™) for Medial Osteochondral Lesions of the Talus: A Prospective One-Year Follow-Up Study
Source: J Clin Med. 2025 Dec 9;14(24):8710. doi: 10.3390/jcm14248710 (PMC12733586; doi:10.3390/jcm14248710)
Supplement: Supplementary file 1 [file jcm-14-08710-s001.zip › jcm-4017315-supplementary.pdf]

Supplemental Table S1. Comparison of patients with and without one-year follow-up.

	**One year follow-up questionnaire available**		
**Characteristic**	**no**<br>**N = 17**[1]	**yes**<br>**N = 12**[1]	**p-value**[2]
Gender			0.5
F	9 (53%)	5 (42%)	
M	8 (47%)	7 (58%)	
Age at treatment			0.13
N Non-missing	17	12	
Mean (SD)	32.8 (15.1)	42.8 (16.7)	
Median (Q1, Q3)	29.0 (20.0, 46.0)	44.0 (31.0, 55.0)	
Min, Max	14.0, 60.0	14.0, 68.0	
Body height [cm]			0.9
N Non-missing	13	10	
Mean (SD)	171.3 (10.8)	169.4 (10.0)	
Median (Q1, Q3)	172.0 (164.0, 174.0)	171.0 (160.0, 176.0)	
Min, Max	150.0, 189.0	152.0, 186.0	
Unknown	4	2	
Body weight [kg]			0.6
N Non-missing	13	10	
Mean (SD)	80.8 (14.4)	77.4 (16.9)	
Median (Q1, Q3)	84.0 (70.0, 90.0)	77.5 (62.0, 89.0)	
Min, Max	54.0, 100.0	54.0, 102.0	
Unknown	4	2	
Body mass index (BMI)			>0.9
N Non-missing	13	10	
Mean (SD)	27.5 (4.7)	26.8 (4.3)	

	One year follow-up questionnaire available		
**Characteristic**	**no**<br>**N = 17**[1]	**yes**<br>**N = 12**[1]	**p-value**[2]
Median (Q1, Q3)	25.5 (23.7, 31.6)	26.4 (25.7, 26.8)	
Min, Max	22.0, 35.9	20.8, 34.5	
Unknown	4	2	
Body mass category			0.2
normal weight	6 (46%)	2 (20%)	
obese	4 (31%)	2 (20%)	
overweight	3 (23%)	6 (60%)	
Unknown	4	2	
Pre-treatment : Visual Analog Pain Scale : Visual Analog Pain Scale			>0.9
N Non-missing	14	11	
Mean (SD)	3.9 (2.6)	3.8 (2.2)	
Median (Q1, Q3)	3.5 (1.8, 6.9)	4.1 (1.4, 5.8)	
Min, Max	0.0, 8.1	0.6, 7.0	
Unknown	3	1	
2 weeks : Visual Analog Pain Scale : Visual Analog Pain Scale			0.8
N Non-missing	13	11	
Mean (SD)	2.7 (1.7)	2.6 (2.0)	
Median (Q1, Q3)	3.0 (1.5, 4.1)	2.1 (0.7, 4.8)	
Min, Max	0.0, 5.1	0.2, 6.0	
Unknown	4	1	
3 months : Visual Analog Pain Scale : Visual Analog Pain Scale			>0.9
N Non-missing	13	12	
Mean (SD)	2.7 (2.1)	2.6 (1.2)	
Median (Q1, Q3)	2.8 (0.9, 4.0)	2.8 (1.6, 3.1)	

	One year follow-up questionnaire available		
**Characteristic**	**no**<br>**N = 17**[1]	**yes**<br>**N = 12**[1]	**p-value**[2]
Min, Max	0.0, 7.0	0.7, 4.5	
Unknown	4	0	
6 months : Visual Analog Pain Scale : Visual Analog Pain Scale			0.2
N Non-missing	11	11	
Mean (SD)	3.9 (3.3)	1.7 (1.3)	
Median (Q1, Q3)	1.5 (0.9, 7.0)	1.4 (0.3, 3.2)	
Min, Max	0.6, 8.2	0.0, 3.6	
Unknown	6	1	
Pre-treatment : VR-12 Physical Score			0.6
N Non-missing	14	11	
Mean (SD)	39.7 (9.5)	37.2 (9.4)	
Median (Q1, Q3)	39.3 (32.4, 47.6)	37.9 (26.8, 43.2)	
Min, Max	22.3, 53.4	25.0, 53.3	
Unknown	3	1	
6 months : VR-12 Physical Score			0.5
N Non-missing	11	11	
Mean (SD)	41.3 (9.0)	44.3 (10.3)	
Median (Q1, Q3)	40.4 (36.2, 50.0)	45.8 (34.9, 53.3)	
Min, Max	26.9, 53.9	26.5, 57.8	
Unknown	6	1	
Pre-treatment : VR-12 Mental Score			0.13
N Non-missing	14	11	
Mean (SD)	47.8 (9.3)	42.5 (9.3)	
Median (Q1, Q3)	48.3 (43.2, 53.3)	39.1 (37.9, 47.5)	
Min, Max	27.7, 62.1	25.0, 57.6	

	One year follow-up questionnaire available		
**Characteristic**	**no**<br>**N = 17**[1]	**yes**<br>**N = 12**[1]	**p-value**[2]
Unknown	3	1	
6 months : VR-12 Mental Score			0.8
N Non-missing	11	11	
Mean (SD)	46.8 (12.4)	48.7 (11.0)	
Median (Q1, Q3)	52.6 (36.0, 57.0)	48.9 (42.3, 59.3)	
Min, Max	24.4, 58.5	30.7, 63.4	
Unknown	6	1	
Pre-treatment : Foot Function Index			0.3
N Non-missing	14	11	
Mean (SD)	31.3 (11.2)	36.7 (12.0)	
Median (Q1, Q3)	33.9 (20.8, 38.3)	38.2 (29.0, 45.5)	
Min, Max	13.2, 47.8	14.4, 58.3	
Unknown	3	1	
3 months : Foot Function Index			>0.9
N Non-missing	13	12	
Mean (SD)	35.3 (14.8)	36.3 (11.4)	
Median (Q1, Q3)	35.8 (22.1, 48.5)	37.5 (30.6, 43.2)	
Min, Max	11.7, 53.2	11.8, 53.3	
Unknown	4	0	
6 months : Foot Function Index			0.3
N Non-missing	11	11	
Mean (SD)	30.4 (19.4)	21.5 (17.6)	
Median (Q1, Q3)	29.4 (12.5, 46.2)	20.0 (6.6, 35.0)	
Min, Max	4.2, 64.7	0.0, 56.7	
Unknown	6	1	
Pre-treatment : FAAM Sports Subscale			0.3

	One year follow-up questionnaire available		
**Characteristic**	**no**<br>**N = 17**[1]	**yes**<br>**N = 12**[1]	**p-value**[2]
N Non-missing	14	11	
Mean (SD)	29.0 (19.4)	34.7 (21.9)	
Median (Q1, Q3)	25.0 (15.6, 34.4)	40.6 (12.5, 43.8)	
Min, Max	6.3, 81.3	0.0, 68.8	
Unknown	3	1	
3 months : FAAM Sports Subscale			0.2
N Non-missing	13	12	
Mean (SD)	24.5 (20.1)	35.7 (23.1)	
Median (Q1, Q3)	18.8 (12.5, 31.3)	39.1 (18.8, 46.9)	
Min, Max	0.0, 75.0	3.1, 81.3	
Unknown	4	0	
6 months : FAAM Sports Subscale			0.014
N Non-missing	11	11	
Mean (SD)	23.0 (23.4)	54.3 (29.4)	
Median (Q1, Q3)	15.6 (6.3, 28.1)	53.1 (25.0, 81.3)	
Min, Max	0.0, 78.1	12.5, 100.0	
Unknown	6	1	

[1]n (%)

[2]Pearson's Chi-squared test; Wilcoxon rank sum test; Wilcoxon rank sum exact test; Fisher's exact test
